# Supplementary material for: A Systematic Review on the Impact of the Social Confinement on People with Autism Spectrum Disorder and Their Caregivers during the COVID-19 Pandemic
Source: Brain Sci. 2021 Oct 22;11(11):1389. doi: 10.3390/brainsci11111389 (PMC8615555; doi:10.3390/brainsci11111389)
Supplement: Supplementary file 1 [file brainsci-11-01389-s001.zip › brainsci-1408399-supplementary.pdf]

Table S1. Description of the characteristics of the 17 analyzed studies

Authors	Country	Study design	Sample size & characteristics	Methods	Focus	Standards Assessment tools
**Physical Activity**						
[51] Exentürk, 2020	Turkey	Qualitative	10 parents	Semi-structured interviews phone	Parents' perceptions on physical activity	
[52] Garcia et al., 2020	USA	Descriptive	9 adolescents	Digital Survey	ASD subjects	
[53] Pfeiffer, y otros, 2021)	USA	Descriptive	6 young adults with ASD	GPS methods and daily participant questionnaires	Changes in activity space use and community mobility	GPS-enabled cell phones
**Psychological Distress among parents of children with ASD**						
[54] Colizzi et al., 2020	Italy	Descriptive	529 parents	Digital Survey	Parents wellbeing and needs	
[55] Manning et al., 2020	USA	Descriptive	471 respondents	Digital Survey	Levels of stress and disruption to life	
[56] Alhuzimi, 2021	Saudi Arabia	Descriptive	150 parents ASD children < 18 years old	e-mail Survey	Stress and emotional well-being of parents	PSI/SF [57]<br>GHQ-12 [58]
[59] Althiabi, 2021	Saudi Arabia	Two groups (with and Without ASD children)	211 parents<br>With children < 18 years old	Digital Survey	Attitude, anxiety and perceived mental health care needs	FIQ [60]<br>HADS [61]<br>GHQ-12[58]
[62] Wang et al., 2021	China	Two groups (with and Without ASD children)	1764 parents ASD children<br>4962 parents Typical Development	Digital Survey	Psychological distress parents	CD-RISC [63]<br>CSQ [64]<br>SAS [65]<br>SDS [66]
[67] Meral, 2021	Turkey	Descriptive qualitative and quantitative	32 parents ASD children	synchronous video call	Quality of live	BFDS [68]<br>FQOL [69]
**Impact of containment and mitigation measures on children and youth with ASD**						
[26] Amorim et al., 2020	Portugal	Descriptive	99 parents<br>43 ASD children<br>56 TD children	Digital Survey	Children	Author's elaboration
[70] Jeste et al., 2020	USA	Descriptive	818 caregiver	Digital Survey	Changes in access to educational and healthcare services	

[71] Mutluer et al., 2020	Turkey	Descriptive and comparison of behaviours before and after the confinement period	87 children and young adults (3-29 years)	Patients data base collection and Digital Survey	Study of the specific challenges generated for pandemic Covid-19 to individuals with ASD and their caregivers	ABC [72]<br>PSQI [73]<br>BAI [74]
[75] Berard et al., 2021	France	Descriptive cross-sectional	239 parents ELENA [77] cohort 2-21 years old	Digital Survey	Children	COVID-19 questionnaire
[77] Lugo-Marín et al., 2021	Spain	Descriptive	37 Childrens<br>35 adults	Digital Survey	Children<br>Adults	CBCL [78]<br>SCL-90-R [79]
[80] Mumbardó-Adam et al., 2021	Spain	Descriptive	47 Parents ASD children	Semi-structured online survey	Children/ family management quarantine	
[81] White et al., 2021	USA	Descriptive	70 faces	Anonymous online survey	Tele-health services	
[82] Siracusano et al., 2021	Italy	Descriptive and comparison of behaviours before and after the confinement period	85 children	Clinical database of the Child Psychiatry Unit of the University of Rome Tor Vergata Hospital	Adaptive functioning, behavioral problems, and repetitive behaviors	ABAS-II [83]<br>RBS-R[84]<br>CBCL [78]
